# Improving continuity of care in Finnish primary health care: Insights from a nationwide qualitative study of primary care physicians

**DOI:** 10.1080/13814788.2025.2583546

**Published:** 2025-11-21

**Authors:** Ulla Mikkonen, Kadri Suija, Tuomas Koskela, Pekka Mäntyselkä, Nina Tusa

**Affiliations:** ^a^ Institute of Public Health and Clinical Nutrition, University of Eastern Finland, Kuopio, Finland; ^b^ Wellbeing Services County of North Savo, Kuopio, Finland; ^c^ Institute of Family Medicine and Public Health, Faculty of Medicine, University of Tartu, Tartu, Estonia; ^d^ Department of General Practice, Faculty of Medicine and Health Technology, Tampere University, Tampere, Finland; ^e^ The Wellbeing Services County of Pirkanmaa, Tampere, Finland

**Keywords:** Continuity of care, primary care, general practitioner, qualitative research, practice management

## Abstract

**Background:**

Continuity of care (COC) is a core value in general practice. It has been deteriorating in Finnish primary health care, but there are now attempts to improve it.

**Objectives:**

This study focused on gathering insights of primary care physicians (PCPs) on how COC can be improved within Finnish primary health care.

**Methods:**

We conducted a nationwide survey targeting all PCPs working in primary health care. A web-based questionnaire was sent to PCPs in Finland from May to October of 2023. The questionnaire included the question ‘How would you improve continuity of care in your workplace?’ Data were analysed using a descriptive approach that involved iterative and inductive thematic analysis.

**Results:**

We received a total of 291 responses from PCPs across Finland (7% response rate). We identified themes related to organisation (e.g. arrangement of practical work including the size of a health centre), practice-level (e.g. autonomy, including the opportunity to perform COC in daily work), and themes related to digital solutions (e.g. data of COC measured and available). PCPs had considerable insight into the development of COC in day-to-day operations.

**Conclusion(s):**

Enhancing COC for a primary care patient population requires a systemic perspective and structured, goal-oriented development efforts. However, small and discrete steps can also contribute to improving COC for individual patients. Our findings highlight the link between COC enhancement and the development of the health care system as a whole.

## KEY MESSAGES

Improving continuity of care is closely linked to the overall development of the health care system

This study proposes organisational, practice-level, and digital strategies to improve continuity of care

Primary care physicians can contribute significantly, drawing on their clinical experience and systemic understanding of health care

## Introduction

Continuity of care (COC) is a core value in general practice [1]. Furthermore, the scientific evidence supporting the implementation of COC in general practice is strong [2–7]. COC is commonly defined in terms of relational, informational, and management COC [8,9]. While the knowledge of the benefits of COC for patients, health care professionals, and health care system is well-established [3–6,10,11], COC has declined in the Finnish health care system. National statistics also indicate a marked decline in COC in recent years [12]. This may be due to the prioritisation of access to care and the reduction of resources at the expense of COC [13].

**Figure 1.**
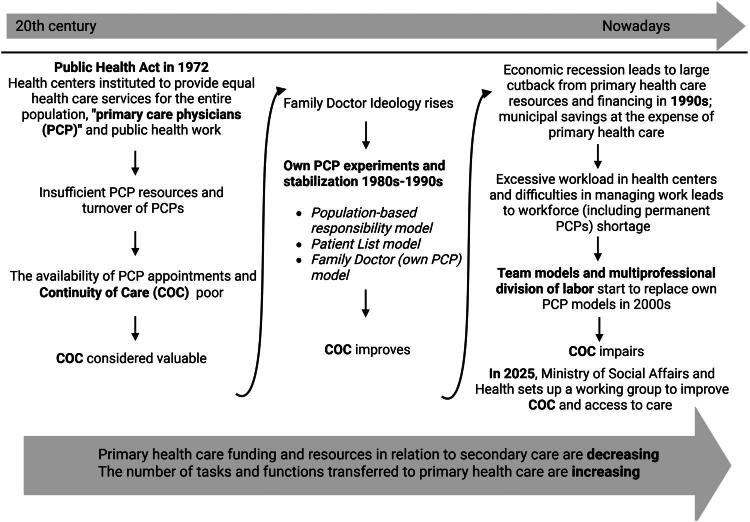
A rough description of the phases of continuity of care in Finnish primary health care. The figure is constructed based on [13].

Changes are taking place in the provision of primary health care worldwide; the expectations of patients and employees are diverse; and digitalisation is expanding in various ways. Efforts to improve system productivity—such as enhancing digital infrastructure—coincide with patient-related factors like complex illnesses and diverse needs, as well as financial challenges. Together with cost-saving pressures, these factors make it increasingly demanding to deliver care that is accessible, patient-centered, and equitable. In a system struggling with burnout, it is challenging to carry out and maintain care in accordance with the core values of general practice that emphasise continuity of doctor-patient relationships as a central organising principle [14]. The primary health care system in Finland has undergone significant changes (Figure 1) that compromise the ability to maintain COC [13,15]. Finnish public health care is primarily tax-funded. The public sector both finances and provides most services. Adult patients pay service fees, but these are capped annually. Primary care physicians (PCPs) at a health centre typically receive either a fixed salary or a combination of base pay and performance-based compensation, but there are no specific incentives for ordering blood tests or other diagnostic procedures.

Only a limited number of studies have investigated the perspectives of PCPs on how to improve COC in current primary health care. In a qualitative study in the Netherlands, the staff and patients perceived relational COC as existing more between a patient and the entire general practice team than between the patient and their own PCP. Thus, improving COC was associated, for example, with effective team communication and staff stability [16]. In another qualitative study from the Netherlands, PCPs identified numerous areas for development in their daily practice, such as increasing time per consultations, supporting small-scale practices, or delegating tasks to allow regular PCPs more time for complex cases [17]. A qualitative study of patients in Spain described health-system-related (e.g. assignment to a personal PCP), organisation-related (e.g. size of a health centre) and doctor-related elements (e.g. commitment to patient care) that contributed to relational COC [18].

A gap remains in understanding health care professionals’ perspectives on actionable strategies to enhance COC. In this study, we aimed to explore the views of PCPs on ways to improve COC within Finnish primary health care.

## Methods

This study was part of a research project titled Good Practices in Primary Health Care [19]. The aim of the project was to describe existing good practices in primary health care and views of PCPs on areas needing development. (Original questionnaire, Supplemental Material S1) The survey was conducted from May to October of 2023. The questionnaire (via Webropol) was targeted at PCPs working in primary health care settings in Finland. In Finnish primary health care, there are specialists in general practice, doctors in residency training, and other non-specialist physicians working in health centres. The invitation was sent by e-mail to the professors of general practice in the universities of Finland and chief physicians across Finland. They disseminated the invitation to PCPs working within their assigned service areas. An invitation and link to the online questionnaire were also shared on a social media platform (Facebook group) used by PCPs. Participation was voluntary.

The survey was completed without identifiable personal data. We collected quantitative data of the respondents as follows: working area, work experience in primary health care, specialist training phase, and number of PCPs in their health centre. We described these as counts and percentages. This study focused on responses to the open-ended question: ‘How would you improve COC in your workplace?’

### Data analysis

We used a qualitative descriptive approach employing inductive, thematic analysis [20] as the method for analysing the data. The researchers (*n* = 5) independently familiarised themselves with the data. All of them identified codes independently (manually/Atlas.ti) without a previously defined codebook or theoretical framework. Then the codes were discussed together. Next, two researchers (UM, NT) grouped the codes that shared the same meaning and drafted an initial thematic map of themes. The study group had several meetings in which the initial thematic map was discussed with the entire research group. During the process, themes were repeatedly compared to the original material to ensure that they reflected it. We found that the main themes discovered in the answers given by PCPs fall under the following categories: organisational, practice-level, and digital solutions. All the researchers involved agreed on the themes. Finally, we revisited the original material using the codes and compared the emerging themes with the overall message of the data. Some selected responses from the original material were then chosen as illustrative quotations. The study was reported in accordance with the Standards for reporting qualitative research [21]. During the analysis, the members of the group included a doctoral researcher (UM), educational chief physician and researcher (NT), associate professors of general practice (KS, TK) and professor of general practice (PM). All were PCPs and had expertise in qualitative research. Having regular discussions with our team of researchers helped to increase reflexivity and reduce unconscious bias.

## Results

### Study sample

We received a total of 291 responses (ranging from short to long answers with various examples, with the data consisting of 6,035 words in total) to the question on improving COC from PCPs across primary health care centres in Finland (Table 1). There are approximately 4,000 vacancies in public primary health care centres across Finland, with an average response rate of 7%.

**Table 1.** Characteristics of respondents (*n* = 291).

	N (%)
**Respondents**	291 (100)
**Working experience in PHC**	
Less than 1 year	6 (2)
1–5 years	60 (21)
Over 5 years	225 (77)
**Education**	
GP specialist	189 (65)
GP trainee	70 (24)
Specialist in other speciality	6 (2)
Trainee in other speciality	3 (1)
Physician in specific training phase^a^	12 (4)
Other physician	11 (4)
**Working area**	
Southern-Finland	76 (26)
Eastern-Finland	87 (30)
Northern-Finland	28 (10)
Inner-Finland	56 (19)
Western-Finland	39 (13)
Other	5 (2)
**Number of PCPs in health centre**	
Less than four physicians	37 (12)
5–10 physicians	57 (20)
More than 10 physicians	196 (68)

^a^Specific Training in General Medical Practice.

PHC: Primary Health Care; PCP: Primary Care Physician.

**Figure 2.**
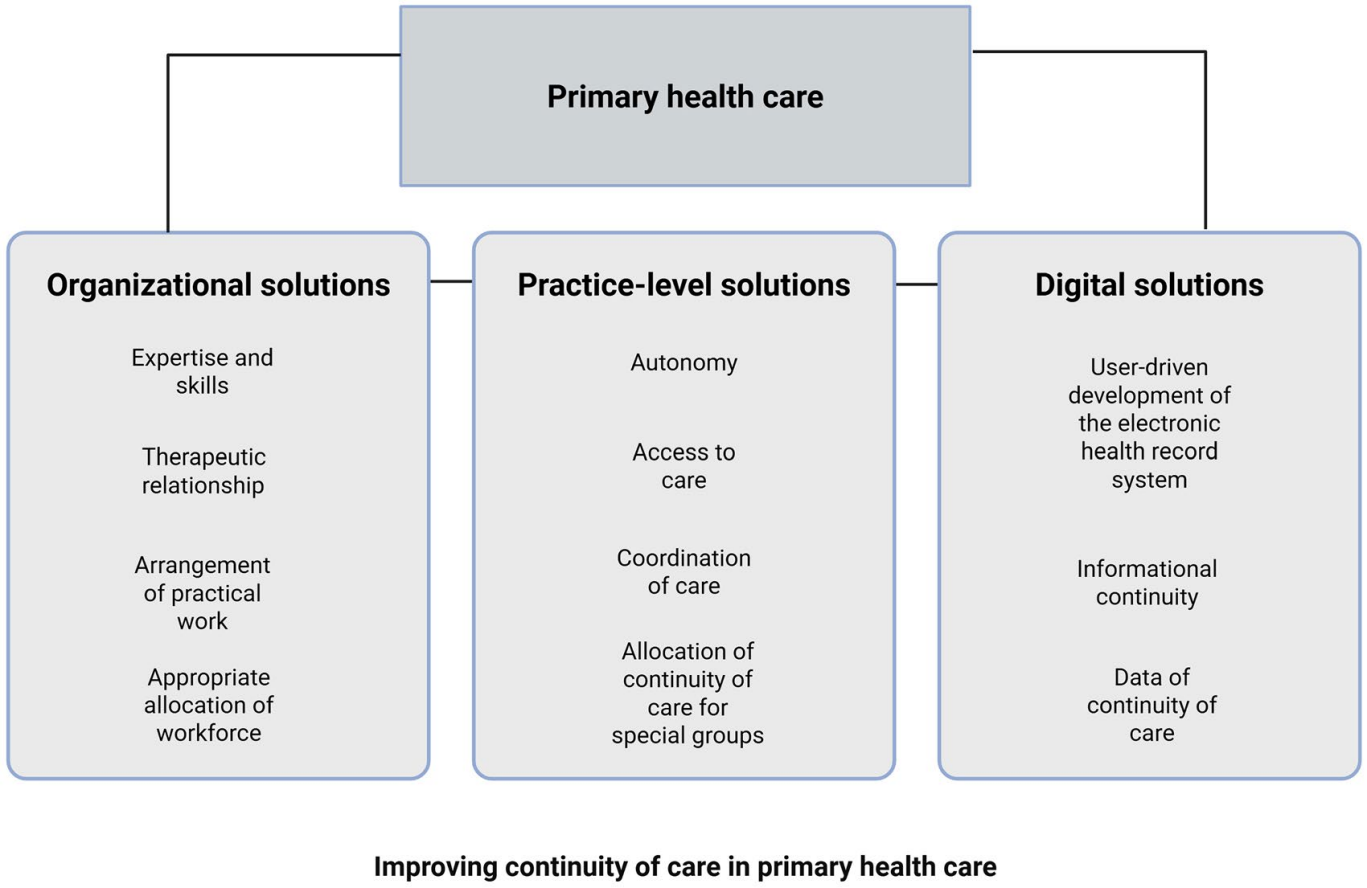
Main themes (*n* = 11) and categories (organisational, practice-level and digital) of improving continuity of care as reported by primary care physicians in Finland.

The themes are illustrated in Figure 2 under organisational, practice-level, and digital solutions. Table 2 presents the main themes (*n* = 11) and subthemes (*n* = 25) with some illustrative quotes. Every quotation is followed by an indication of whether the respondent was a specialist in general practice (S), resident doctor (R), or other PCP (A) as well as years of working experience in primary health care. The following sections describe the results of each main theme.

**Table 2.** The results of thematic analysis based on data of 291 primary care physicians in Finland.

Level	Main theme	Subthemes	Quotation examples
Organisational solutions	Therapeutic relationship	Relational continuity is considered in policy making Enabling patient-centred care	*‘Continuity of care should be number one when the system is being fine-tuned. That is, the entire staff should recognize its importance, in which case it would guide their actions.’* *(O422:* **S, > 5 years)** **‘***Patients should be able to choose their own PCP, that would be an ideal situation’* *(O61:* **S, > 5 years)**
	Expertise and skills	Physicians’ level of training and professional experience is considered in the division of tasks Training for health care professionals of the assessment of patient’s need for services	*‘The training system does not support COC, because young doctors come to the health centre for 6–9-month periods and they represent up to two-thirds of all doctors. Senior doctors have supervision and various other responsibilities and consultations, so the number of patients they have decreases.’* *(O24:* **S, > 5 years)** *‘On the other hand, more nurses would also be needed or should be trained more in assessing the need for care, so that they would have more time or expertise to assess and plan the patient’s further care.’* *(O100:* ***R, < 1 year)***
	Appropriate allocation of workforce	Rational use of resources Supplementary services Collaborative and integrated services	*‘When outsourcing service providers are used, I would like them to take care of the patient’s matter as a whole process, rather than having to assess the treatment of the same issue later at a (public) health centre (such as the interpretation of lab results) as well.’* *(O163:* **S, > 5 years)** *‘In mental health services at primary health care, there should be an aim away from short, rigid treatment periods and prioritise long-term follow-up of long-term diagnoses by the same team*.’ (O64: **S, > 5 years)** *‘A team model for everyone who uses a lot of services and I would strengthen the integration of social and health care services.’* *(O312:* **S, > 5 years)**
	Arrangement of practical work	Different models to arrange patient care (team-based, own PCP etc.) Size of health centre	*‘An experienced doctor has, for example, a population of 2,500, plus a young doctor. Together they treat the population in a specific area.’* *(O24:* **S, > 5 years)** *‘ The challenge of COC is an inadequate and changing workforce. The working conditions must be such that people enjoy working longer.’* *(O427:* **S, > 5 years)** *‘Somewhat towards an own PCP model, but I would still prefer a more flexible system. I hope the units remain reasonably small and that physician satisfaction is high enough to foster long-term patient relationships. Shouldn’t there also be room for career development while still being able to maintain continuity in patient care?’* *(O232:* **S, > 5 years)**
Practice-level solutions	Autonomy	Opportunity to perform continuity of care in daily work Possibility to resolve practical issues locally	*‘PCPs should have the possibility to program the system so that the results of a patient’s examination go to their own list to be viewed. For example, it should be possible for the PCP to book call-back times etc. to discuss the results, so that they (the patients) would not have to go see just any available doctor. There should be time for office work or phone calls etc. that can be reserved for handling these issues.’* *(O392:* ***S, > 5 years)*** *‘We should be allowed to maintain and develop good and proven practices at the local level.’* *(O10:* **S, > 5 years)**
	Access to care	Assessing the patient’s care needs by familiar professionals The patient’s own PCP helps to assess the care needs Patients are always referred to their own PCP PCP can book further consultations directly at themselves Using electronic invitation system to organise proactive and systematic care of/for chronic diseases	*‘I would dismantle the centralised calling centres. Appointments should be carried out in smaller units, allowing both nurses and doctors to get to know the patients.’* *(O225:* **S, > 5 years)** *‘I think it would be important for the patient to initially be referred to the same doctor if he or she is at work at the time.’* *(O14:* ***R, >5 years)*** *‘More frequent consultations with one’s own PCP, without automatically scheduling an appointment—the PCP might have other solutions.’* *(O364:* ***R, 1–5 years)*** *‘Treatment of long-term diseases should be carried in one team using an electronic invitation system and having a multi-professional division of work and modelling ways to work, taking into account resources.’* *(O126:* ***S, > 5 years)***
	Allocation of continuity of care for special groups	Identification of people who are multimorbid or overuse healthcare	*‘Patients who need a lot of contacts with health services need contacts from a familiar nurse. For example, a doctor, a nurse, and a patient could agree that the nurse would be in contact with the patient as often as once every two weeks, and then if something new comes up, either a consultation or a doctor’s contact with the patient. In this way, the patient could be supported without the need for numerous acute visits, failure demand, yet in such a way that more time would be freed up for the doctor for the treatment of other patients.’* *(O291:* **S, > 5 years)** *‘Not everyone needs the same person (doctor). In episodic care, the next contact should be matched to the same doctor. In the case of long-term diseases, the patient should have his or her own PCP if there have been challenges in the treatment.’* *(O198:* **S, > 5 years)**
	Coordination of care	Remote consultations are referred to the one’s own GP Care pathways and common practices support continuity of care	*‘The so-called team model should be dismantled. In the team model, the nurses call the patient, and pass information to the doctor, who enters something into the electronic health system and the nurse calls the patient again, and, for example, the lab results are then looked at by another nurse, who asks for a comment on the results from another doctor, whose comments the nurse then relays to the patient by phone, etc., an endless broken telephone system.’* *(O380:* **S, > 5 years)** *‘By clarifying care pathways, we can reduce the unnecessary ‘bobbing around’ that patients currently experience in the system.’* *(O179:* ***A, 1–5 years)***
Digital solutions	User-driven development of the electronic health record system	The same electronic health record system for all care providers Electronic health record systems support continuity of care	*‘The same electronic health record system for the whole well-being area and preferably for the whole of Finland. A Finnish patient information system would be produced, serving users and operating on their terms. The producer of the electronic health record system would make corrections and changes on the terms of the users, not so that the producer dictates how much a thing costs and add-ons or repairs cost more. Many municipalities had to change the electronic health record system when the upgrade and maintenance to the new version was too expensive.’* *(O173:* ***R, 1–5 years)*** *‘An electronic system to replace human work. At least in the case of annual check-ups, it would be relatively easy to implement, and patients often neglect to reserve these (annual check-ups) for themselves. A diabetic with poor blood sugar levels or a coronary artery disease patient with poor cholesterol and blood pressure values often renews prescriptions year after year without check-ups.’* *(O178:* **S, > 5 years)**
	Information continuity	Conjointly updated medication lists, care plans, treatment goals and functional capacity recorded and available Digital tools serving and supporting continuity of care	*‘If the patient is well-treated for chronic diseases and has a care plan, even acute situations could be handled by a consultation with his or her own PCP. The basic preliminary information and updated medication list should be recorded in electronic health records and be easy to find.’* *(O166:* **S, > 5 years)** *‘Patients would benefit from a summary in the electronic health system that shows which doctors should handle the patients’ cases and to whom appointments can be given. This is not always clear when the nurses assess care needs.’* *(O282:* ***R, <1 year)***
	Data of continuity of care	Measuring continuity of care Shared information, goal and engagement to improve continuity of care	*‘It should be possible to monitor the COC of each doctor and team in the statistics. Different teams within the same health centre can work very differently, making it difficult to receive information about the COC of one’s own team.’* *(O163:* **S, > 5 years)** *‘The leaders have adopted COCI, which is good. But no goals have been set for it, and no management decision supports COC. This should be developed. COC should be actively monitored and a target set for the well-being area as well. COC is based only on the activity of one’s own team. We must demand that all health centres develop practices that support COC, and if there is no COC, we must talk about it and rethink ways to develop it.’* *(O163:* **S, > 5 years)**

S: Specialist in general practice; R: Resident doctor; PCP: Primary care physician; A: Another doctor; COC: Continuity of care.

#### Therapeutic relationship

PCPs mentioned that policymakers should understand the importance of COC in decision-making and its importance for patients’ treatment results. Preserving patients’ ability to choose their own PCP was considered important.

#### Expertise and skills

PCPs stated that a physician’s level of training and work experience should be considered when allocating tasks and responsibilities. The number of experienced PCPs performing patient work was seen as important, and the respondents felt that it is not reasonable to assign a patient population to a physician who is doing a brief period of work. Instead, they should jointly take care of the same population with a specialist in general practice. COC was seen as producing valuable information for resident doctors regarding their work quality and learning.

#### Appropriate allocation of workforce

For PCPs, the rational use of resources means that each professional group performs tasks aligned with its specific expertise and level of education. The workforce of experienced PCPs was seen as important for maintaining longitudinal care relationships in clinical practice.

Health services that complement public health care (supplementary services), such as occupational health care or outsourcing service providers, should take care of the patient’s entire care process without fragmentation of care. Similarly, remote consultations should support COC.

Collaboration between different occupational groups and care providers, as well as between social welfare and health care, should be promoted. Organisational structures should enable the comprehensive, rather than siloed, care of the patient.

#### Arrangement of practical work

PCPs called for sufficient resources. The respondents identified different models to organise longitudinal therapeutic relationships, such as the patient’s own PCP-based model, a team-based model, or a model involving a pair of an experienced PCP (specialist in general practice) and a resident doctor. The small size of a health centre was seen as a factor that promoted familiarity between patients and health centre staff. They also proposed that a substitute should be appointed during PCPs’ holidays.

#### Autonomy

Autonomy was described by PCPs as managing one’s own schedule and having enough discretionary time to provide consultations based on the needs of their own population. Autonomy also included the opportunity for those engaged in practical work to participate in planning and organising their daily tasks. Furthermore, they should have assigned work time for development tasks.

#### Access to care

PCPs stated that the assessment of care needs should be carried out by professionals who are already familiar with the patients and population. To assess whether an appointment is required for the patient, or whether the problem can be resolved in an alternative manner, consultation with the patient’s own PCP is recommended. An electronic invitation system could be used to organise the treatment of long-term diseases timely and to refer patients to their own PCP.

#### Allocation of COC for special groups

PCPs recognised that at least multimorbid patients and high health care utilisers should have their own PCP or case manager. However, patient allocation should be coordinated in order to prevent excessive workload on individual PCPs. Respondents also felt that more knowledge is still needed regarding patients who do not benefit from continuity.

#### Coordination of care

Interprofessional consultation in patient care should be easy to implement and seamlessly integrated into daily practice. Remote and digital consultations, like other forms of patient care, should be applied appropriately to ensure quality and effectiveness. Common care paths and practices support the smoothest possible care.

#### User-driven development of the electronic health record system (EHRS)

PCPs proposed an EHRS that could identify and recommend a PCP/responsible employee when the patient schedules an appointment. When a patient renews a prescription, the system would alert them if annual check-ups have not been completed. The system should send an alert if the patient has poor control of long-term conditions or risks identified in the course of primary prevention.

#### Informational continuity

PCPs considered important that primary and secondary care staff update patients’ medication lists, care plans and treatment goals, along with a description of their functional capacity in the EHRS so that these data are available for all involved in the care process. PCPs suggested that digital tools could be used for supporting relational COC and collecting preliminary data before an appointment (questionnaires, health behaviours etc.).

#### Data of COC

Respondents considered it important that COC be set as a shared goal in health care. COC should be measured regularly, and information should be available to employees to get feedback on the quality of their work.

## Discussion

### Main findings

PCPs identified several elements that support COC in primary health care. Enhancing COC for a broad patient population requires a systemic perspective and structured, goal-oriented development efforts such as enhancing sufficient resources in primary health care, using the workforce appropriately, preserving PCPs’ autonomy in managing their daily work, and improving collaborative and integrated services. However, multiple small, discrete actions such as updating medication lists, referring patients to their own PCP or using an electronic invitation system to organise follow-up care for long-term conditions could also improve COC for individual patients.

### Comparison with existing literature

Based on our results, it is crucial to maintain sufficient resources in primary health care, taking into account holiday and training periods of permanent staff. A recent analysis considered organisational aspects in implementing COC in the UK. The authors stated that to increase COC, the whole practice should be committed to the project. A shared goal should be targeted and measured. As a result, health care professionals and patients receive feedback on the potential benefits. If not properly managed, responsibility may shift to individual professionals, leading to a decline in COC [22]. PCPs in our study also perceived that COC should be measured and this information utilised in day-to-day management.

In our study, solutions to improve access to care such as the centralisation of services into large-scale units and the implementation of team-based models—both of which have been proposed to address the shortage of health care personnel in Finland—were found to pose challenges for COC. Interestingly, another study suggested that team-based care might provide the means to maintain relational COC. However, the authors also felt that enabling this would likely require that teams remain small and consist of stable personnel, and it is essential to consider how tasks are distributed among different professional groups [16]. The PCPs in our study emphasised the importance of preserving the ability to flexibly adjust daily operations at the local level. Development of COC in the system requires joint dialogue, goal setting and follow-up monitoring at the local level [23].

In Finland, participation in specialist medical training contributes to turnover within the primary care workforce. All specialising doctors are required to work in primary health care for varying mandatory periods. Thus, the resources of PCPs will continue to be variable and pose challenges to relational COC in the future. Earlier proposals have suggested a pair of PCPs working together to ensure relational COC [16]. Similarly, our data suggest that relational COC could be distributed within smaller teams, such as pairing a specialist with a resident physician to jointly manage care. In addition, the number of part-time PCPs is increasing. However, there is also some evidence that clinic-specific COC (seeing own PCP or if not available, another PCP in the same clinic) is associated with less use of emergency services or need for hospital care [24].

One aspect in developing COC is the preferences of patients. Some prefer fast access to care instead of COC. This preference may also vary for the same individual depending on the problem for which they are seeking help [25]. In our study, the PCPs also discussed which patients should be targeted for COC, which is in line with previous qualitative studies [16,26]. However, it is important to remember that the individuals who would benefit most from COC or services do not always ask for or receive them [27].

In our study, PCPs proposed multiple digital solutions that could improve COC. In a qualitative study in Belgium, shared health records were perceived as supporting interprofessional team work in general practice [28]. In another study, respondents also noted that more effective use of EHRS could improve informational COC in team communication [16]. In addition, the EHRS could aid staff in scheduling appointments with the same PCP [17]. By improving informational and management COC, it may be possible to partially offset the disadvantages of a disrupted relational COC [5]. A recent Finnish study suggested that doctors can experience stress when digital systems do not support their work [29]. Well-functioning systems can support informational COC as well as the comfort and retention of doctors, and thus COC.

Efforts have been made in Finland to resolve the dilemma between the need, demand, and availability of services in different ways, such as by increasing multiprofessional team-based work. Maintenance of everyday clinical work solutions that support COC requires that staff perceive it as an important and shared goal. When the staff is volatile and there is also a widespread shortage of PCPs in Europe [30], it is important to find new solutions to maintain the core values of general practice in clinical practice. However, the implementation of new clinical practices requires time as well as adequate support structures and sufficient resources [26]. In summary, the clinical practice proposals presented in this study demonstrate an appreciation of functional teamwork to provide patients with a seamless and appropriate care pathway.

#### Strengths and limitations

The study produced perspectives for improving COC. The responses were mainly from specialists in general practice or junior doctors in residency training in general practice, offering a rich and diverse perspective. Responses were from all areas of Finland. In addition, most of them had extensive working experience in primary health care. The respondents produced a wealth of answers to open-ended questions, which indicates the importance of the topic for them. A strength of the study was the use of several methods to share the invitation link.

Answers to the open-ended questions were received in written form and some of them were short. It was not possible to make a specific interpretation of the answers from the respondents, and the authors’ background factors may have had an impact on interpretation. In this study, doctors willing to participate might hold different views from those who did not. However, the answers of the respondents probably reflect the Finnish health care context. The questionnaire did not specify COC in more detail, which may have influenced the responses. We did not want to focus on just one part of COC, as its dimensions are interconnected.

### Implications for practice and policy

The results contain internationally relevant information, even though health care systems vary. The key question is how to implement these practices. Based on the results, leadership and vision at the organisational level play a key role. Unfortunately, in large organisations, which are common in Finland, the day-to-day management is sometimes handled at a higher level, making it difficult to make changes at the practical level or improve day-to-day operations. Our study highlights the importance of aligning daily work and system management with the practical work environment, alongside shared goal setting, continuous monitoring, and timely feedback.

### In conclusion

Health care is undergoing continuous transformation, necessitating the parallel development of strategies to maintain COC within this evolving landscape. Involving practitioners in the development process is essential, as their firsthand experience provides valuable insight into the operational environment. Such collaboration ensures that the resulting practices remain relevant and applicable, even amid systemic changes. Enhancing COC should not be viewed as an isolated initiative but rather as an integral component of strengthening the primary health care system as a whole.

## Supplementary Material

Supplemental Material

## Data Availability

Written answers in Finnish can be provided upon reasonable request.
