# Supplementary material for: Improving continuity of care in Finnish primary health care: Insights from a nationwide qualitative study of primary care physicians
Source: Eur J Gen Pract. 2025 Nov 21;31(1):2583546. doi: 10.1080/13814788.2025.2583546 (PMC12642884; doi:10.1080/13814788.2025.2583546)
Supplement: Supplemental Material [file IGEN_A_2583546_SM2605.docx]

**Supplement material 1. Good Practices in Primary Health Care – Survey Questionnaire**

1. How long working experience do you have in primary health care?
   1. Less than a year
   2. 1-5 years
   3. More than 5 years
2. You are
   1. Specialist in general practice
   2. Junior doctor in general practice
   3. Other doctor
3. In which wellbeing county you are working in?
4. You can develop your work
   1. Strongly agree
   2. Slightly agree
   3. Slightly disagree
   4. Strongly disagree
5. If you have had the opportunity to develop your work, please describe how? In your response, you may describe both the development of your personal work and other developments in primary health care. **(Open-ended)**
6. If you have had the opportunity to expand your skills and knowledge, please describe how? **(Open-ended)**
7. Patients appreciate your work
   1. Strongly agree
   2. Slightly agree
   3. Slightly disagree
   4. Strongly disagree
8. Work community appreciate your work
   1. Strongly agree
   2. Slightly agree
   3. Slightly disagree
   4. Strongly disagree
9. Leaders appreciate your work
   1. Strongly agree
   2. Slightly agree
   3. Slightly disagree
   4. Strongly disagree
10. Continuity of care is realized at your workplace
    1. Strongly agree
    2. Slightly agree
    3. Slightly disagree
    4. Strongly disagree
11. Please describe good practices that have enabled continuity of care at your workplace **(Open-ended)**
12. How would you improve continuity of care at your workplace? **(Open-ended)**
13. At your workplace, care can be provided in a patient-centred manner, meaning that the patient is at the centre of care and their care pathway is as fluent as possible.
    1. Strongly agree
    2. Slightly agree
    3. Slightly disagree
    4. Strongly disagree
14. At your workplace, patients in need of medical care are appropriately directed to a doctor's appointment
    1. Strongly agree
    2. Slightly agree
    3. Slightly disagree
    4. Strongly disagree
15. Interprofessional collaboration between different professional groups in patient care is appropriate (e.g., social worker, physiotherapist, nurse)
    1. Strongly agree
    2. Slightly agree
    3. Slightly disagree
    4. Strongly disagree
16. Please describe good practices for implementing interprofessional collaboration between different professional groups at your workplace. **(Open-ended)**
17. How would you improve interprofessional collaboration between different professional groups at your workplace? **(Open-ended)**
18. Patient care in collaboration with secondary health care is seamless.
    1. Strongly agree
    2. Slightly agree
    3. Slightly disagree
    4. Strongly disagree
19. Please describe good practices for collaboration between primary health care and secondary health care in your area. **(Open-ended)**
20. How would you improve collaboration with secondary health care? **(Open-ended)**
21. Work activities at your practice are well organized
    1. Strongly agree
    2. Slightly agree
    3. Slightly disagree
    4. Strongly disagree
22. Please describe good practices for implementing well-organized work activities at your practice. **(Open-ended)**
23. How would you improve work activities in your workplace? **(Open-ended)**
24. Your workplace has good working atmosphere.
    1. Strongly agree
    2. Slightly agree
    3. Slightly disagree
    4. Strongly disagree
25. You can discuss the development of your work with your supervisors/leaders
    1. Strongly agree
    2. Slightly agree
    3. Slightly disagree
    4. Strongly disagree
26. Describe good practices for promoting a positive work atmosphere and collaboration with supervisors/leaders at your workplace. **(Open-ended)**
27. How would you improve the well-being of your work community or collaboration with supervisors/leaders? **(Open-ended)**
28. What would your dream job in primary health care look like? In other words, what should your daily work include so that it aligns with your values? You may also comment on aspects related to job management. **(Open-ended)**
